# Evaluation of the Pharmacokinetic Interactions of *Maytenus ilicifolia* Mart. ex Reiss Extracts on Drug Intestinal Permeability and Hepatic Metabolism

**DOI:** 10.1021/acsomega.5c07564

**Published:** 2025-09-25

**Authors:** Sara Batista do Nascimento, Pedro Henrique Gomes dos Santos, Gustavo Henrique Oliveira Costa, Ícaro Salgado Perovani, Anderson Rodrigo Moraes de Oliveira, José Eduardo Gonçalves, Whocely Victor de Castro, Isabela da Costa César

**Affiliations:** † Departamento de Produtos Farmacêuticos, Faculdade de Farmácia, 28114 Universidade Federal de Minas Gerais, Avenida Presidente Antônio Carlos 6627, Belo Horizonte, MG 31270-901, Brazil; ‡ Departamento de Química, Faculdade de Filosofia, Ciências e Letras de Ribeirão Preto, Universidade de São Paulo, Av. Bandeirantes, 3900, Vila Monte Alegre, Ribeirão Preto, SP 14040-900, Brazil; § Programa de Pós-graduação em Ciências Farmacêuticas, Campus Centro-Oeste Dona Lindu, Universidade Federal de São João del Rei, Rua Sebastião Gonçalvez Coelho, 400, Chanadour, Divinópolis, MG 35501-296, Brazil

## Abstract

*Maytenus ilicifolia* Mart. ex Reiss is a plant native to South America, popularly used in the treatment of gastric disorders. The increase in the consumption of herbal products associated with conventional medications demands attention for the potential risks of interactions. The aim of the study was to evaluate potential pharmacokinetic interactions due to coadministration of *M. ilicifolia* extracts with drug substrates of P-glycoprotein (P-gp) and cytochrome P450 3A4 isoform (CYP3A4). Five extracts of *M. ilicifolia* were prepared, and the contents of the main secondary metabolites were determined. A high-performance liquid chromatography (HPLC) method was developed for the simultaneous quantitation of midazolam, nifedipine, and their respective metabolites and applied to assess the potential of *M. ilicifolia* extracts on hepatic metabolism mediated by the CYP3A4 enzyme. The influence of the extracts on the intestinal permeability of fexofenadine was evaluated by determining P-gp activity, using a Caco-2 cell model. The extracts were characterized in terms of total phenolic (1.77–11.46%), tannin (1.67–3.36%), and flavonoid (0.21–2.64%). The hydroacetonic extract (HAE1) exhibited a remaining activity (%RA) of 50.1% for the 1-hydroxylation of midazolam and 40.1% for the oxidation of nifedipine, indicating moderate inhibition of CYP3A4. HAE1 reduced the fexofenadine efflux ratio to an extent similar to that of verapamil (IC_50_ = 20.42 μg/mL), suggesting an inhibitory effect on the P-gp activity. The extracts demonstrated the potential to inhibit both CYP3A4 and P-gp. Therefore, coadministration of *M. ilicifolia*-based preparations may potentially alter the pharmacokinetics of drugs that are substrates of these systems.

## Graphical abstract

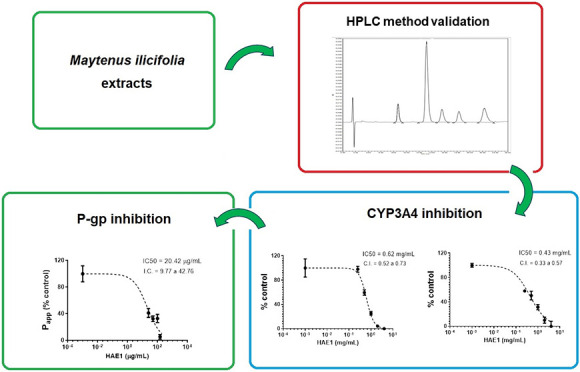

## Introduction

*Maytenus ilicifolia* Mart. ex Reiss (Celastracea), popularly known as Espinheira-santa, is a plant native to South America and widely used to treat gastric disorders.^1^ The leaves, stem bark, and root were widely used by the indigenous population in Brazil for the treatment of urinary tract diseases, diarrhea, intestinal or menstrual cramps, and for the asepsis of wounds.^2^ Its protective effect on the gastric system involves multiple mechanisms of action. One gastroprotective action occurs by inhibition of histamine H2 receptors on parietal cells, thereby reducing acid secretion induced by histamine and gastrin.^3^ In addition, tannins from this plant inhibit the potassium-dependent membrane ATPase in gastric mucosal cells, responsible for the secretion of hydrochloric acid into the stomach.^4,5^ Furthermore, *M. ilicifolia* also exerts bacteriostatic action on *Helicobacter pylori* by altering the permeability of the bacterial membrane and reducing the adhesion of the bacteria to the gastric mucosa.^6^

Phytochemical studies of *M. ilicifolia* have indicated the presence of terpenes (maytenin, tringenone, isotenginone II, mantenoic acid, congorosins A and B), triterpenes (friedelanol, friedelin), chlorogenic acid derivatives (caffeic acid and caffeoylquinic acid isomers), and alkaloids (maitein, maytanprine, maitensine). Flavonoids such as rutin and quercetin, and glycosylated flavonoids have also been described.^7,8^ Condensed tannins were identified as the major chemical constituents in extracts of *M. ilicifolia* and used as markers for quality control of the extracts and herbal products.^9^ The quantitation of catechin, epicatechin, quercetin, kaempferol, and friedelin in several *M. ilicifolia* extracts was described using liquid (LC) and gas chromatography (GC) methods.^10^

Although there is no evidence of toxicity in humans and rodents related to the use of *M. ilicifolia*,^11^ few studies have evaluated the effects of its coadministration with conventional drugs. It is well established that secondary metabolites in plants and foods may affect the pharmacokinetic processes, such as plasma protein-binding, hepatic metabolism, renal excretion, and absorption.^12^ In fact, polyphenols may alter the expression and activity of the CYP3A4 enzymes. Moreover, the flavonoids have been identified as substrates and inhibitors of cytochrome P450 enzymes.^13^ In addition, they can interact with the transmembrane domain of P-glycoprotein (P-gp) in the intestinal epithelium. As result, the drug oral bioavailability can be altered when coadministered with such derivatives, leading to subtherapeutic end points or toxicity.^13,14^ These effects fall under the broader category of herb–drug interactions (HDI), which may result from the induction or inhibition of metabolic enzymes or transport proteins.^15^

A previous study from our group demonstrated that the hydroacetonic and aqueous extracts (AEs) of *M. ilicifolia* were able to double the intracellular concentrations of fexofenadine (a P-gp substrate) in Caco-2 cells, when compared to the control.^16^ Additionally, preclinical tests in male Wistar rats showed that coadministration of *M. ilicifolia* extracts with midazolam (a CYP3A4 substrate) increased in 3-fold the area under the curve (AUC) and 4-fold the maximum plasma concentration (*C*_max_) of the drug.^17^ We hypothesized that the modulation of midazolam pharmacokinetics occurred due to CYP3A4 inhibition. Evaluation of such interactions is essential since they can significantly impair the pharmacokinetics of the affected drug. Inhibition of key metabolic enzymes, like CYP3A4, may reduce presystemic drug elimination, resulting in higher-than-expected bioavailability and consequently, toxic effects.^15^

Thus, the aim of this study was to investigate the influence of *M. ilicifolia* on the CYP3A4 and P-gp activities. An *in vitro* model was used to evaluate the potential of *M. ilicifolia* extracts to inhibit CYP3A4, using human liver microsomes. In addition, the potency (IC_50_) of the plant extracts on P-gp activity was also determined by evaluating the fexofenadine permeability across the Caco-2 cell monolayer.

## Materials and Methods

### Chemicals and Reagents

Midazolam maleate (>99%) was purchased from Fagron (São Paulo, Brazil), 1-hydroxymidazolam (>99%) from Lipomed (Arlesheim, Germany), alprazolam and ketoconazole (>99%) from Pharma Nostra (São Paulo, Brazil), nifedipine (≥98%) and galic acid (>99%) from Sigma-Aldrich (St. Louis, USA), dehydronifedipine (≥98%) from Toronto Research Chemicals (Toronto, Canada), fexofenadine hydrochloride (>99%) from Alekhya (Andhra Pradesh, India), and verapamil hydrochloride (>99%) from Ariston Ltd. (São Paulo, Brazil). The reference standard compounds (>99%) catechin, epicatechin, quercetin, kaempferol, and friedelin, as well as the nicotinamide adenine dinucleotide phosphate sodium salt (NADP^+^), glucose-6-phosphate, glucose-6-phosphate dehydrogenase type VII from yeast, human liver microsomes, hide powder, sodium pyruvate, Dubelcco’s modified Eagle’s medium (DMEM), Hank’s balanced salt solution buffer (HBSS), nonessential amino acids, trypsin solution 0.05%–ethylenediaminetetraacetic acid (EDTA) (0.02%), and penicillin G solution (10 000 U/mL) were all from Sigma-Aldrich (St. Louis, USA). Inactivated fetal bovine serum was obtained from Cultilab (Campinas, Brazil). Transwell plates were purchased from Corning Costar (Cambridge, MA, USA). Methanol and acetonitrile high-performance liquid chromatography (HPLC) grade were from Tedia (Fairfield, OH, USA). Ultrapure water was obtained from a Millipore system (Bedford, MA, USA). All other chemicals were of analytical grade.

### Plant Materials and Extracts

The *M. ilicifolia* leaves were obtained from the Medicinal Plant Garden of the Universidade Federal de Grande Dourados (Dourados, MS, Brazil) at an altitude of 452 m and coordinates 22°11′43.7″ S, 54°56′08.5″ W. The leaves were harvested in winter, in the early hours of the morning, after there was no more dew on their surfaces. The voucher specimen was deposited in the Herbarium of the Universidade Federal de Grande Dourados, under the number DDMS 4882. The leaves (500 g) were dried in an oven at 40 °C and pulverized in a knife mill. Dried leaves of *M. ilicifolia* were also purchased at the Central Market of Belo Horizonte (MG, Brazil), and a commercial dry extract (CDE) was obtained from Florien (São Paulo, Brazil).

Four extracts were produced with these samples using a ratio of 21 g of powder and 1050 mL of solvent. Aqueous extract (AE) was prepared by infusion (80 ± 5 °C) and kept at rest until it reached room temperature. Hydroacetonic extract 1 (HAE1) was obtained using turboextraction at 4000 rpm for 30 min and acetone/water (70:30). Hydroethanolic extract (HEE) was also prepared by turboextraction under the same conditions, using ethanol and water (70:30). All extracts were prepared with the leaves collected at the Medicinal Plant Garden, and a hydroacetonic extract was also obtained from leaves purchased at Central Market (HAE2). The extracts were filtered, concentrated in a rotary evaporator, lyophilized, and stored under refrigeration.

### Determination of Total Phenolics, Tannins, and Flavonoids

The total phenolic content was determined using the Folin–Ciocalteu colorimetric method.^18^ Working solutions of the extracts were prepared in water at a concentration of 0.6 mg/mL. A 0.5 mg/mL gallic acid solution in water was used as a standard. To quantify the total polyphenols, 0.8 mL of the sample or standard solutions, 0.4 mL of Folin–Ciocalteu reagent, and 4 mL of water were transferred to a 10 mL volumetric flask. The volume was completed with a 29% (w/v) sodium carbonate solution and homogenized. Absorbance was measured at 760 nm after 30 min. The total tannin content was determined by the difference between the amount of total phenolics and nontannic phenolics. Sample solutions for nontannic phenolics were prepared by transferring 3 mL of the extract solution at 3 mg/mL and 0.03 g of hide powder to an Erlenmeyer flask and shaking for 60 min. The suspension was filtered, and 1 mL of the suspension was transferred to a 5 mL volumetric flask. The volume was completed with water. Absorbance was measured at 760 nm after 30 min, and the total tannin content was expressed in terms of gallic acid.

The total flavonoid content was determined using the aluminum chloride complexation method. Working solutions of the extracts were prepared in 50% ethanol at 10 mg/mL. A calibration curve was constructed with quercetin in the concentration range from 1.25 to 20 μg/mL. Aliquots of 2 mL of each standard or sample solution were mixed with 500 μL of 5% (w/v) aluminum chloride in test tubes. The mixture was allowed to stand for 30 min, and the absorbance was measured at 425 nm. The flavonoid content was calculated and expressed as the percentage of flavonoids equivalent to quercetin per gram of dry extract. All procedures were performed in triplicate.

### HPLC Method for the Simultaneous Quantitation of Midazolam, 1-Hydroxymidazolam, Nifedipine, and Dehydronifedipine

An HPLC analytical method was developed and validated for the simultaneous quantification of midazolam, nifedipine, and their respective metabolites, used as markers of CYP3A4 activity. The HPLC analyses were carried out on a Waters Alliance system (New Castle, USA), composed of a quaternary pump, autosampler, UV/vis detector, and Empower software. Separation was achieved using a Zorbax Eclipse Plus C18 column (150 mm × 4.6 mm, 5 μm) from Agilent (Santa Clara, USA), at 40 °C. The mobile phase was composed of 10 mM sodium acetate buffer (pH 4.7) and methanol (50:50), with a flow rate of 0.8 mL/min. UV detection was performed at 220 nm, and the injection volume was 50 μL.

For method validation, calibration curves were constructed in methanol, with concentrations ranging from 0.5 to 6.0 μM for midazolam and 1-hydroxymidazolam, and from 0.5 to 8.0 μM for nifedipine and dehydronifedipine. Alprazolam (300 μM) in methanol was used as an internal standard (IS). A linear regression model was employed to define the relationship between the concentration and response of the analytes. Regression analysis was conducted using the ordinary least-squares method following an assessment of normality, independence, and homoscedasticity of the residuals. Linearity was evaluated using the coefficient of determination (*r* ^2^), and analysis of variance (ANOVA) was employed to verify the significance of the regression and deviations from linearity (α = 0.05). The limit of quantification was determined as the lowest concentration on the calibration curve with adequate precision and accuracy.

Precision and accuracy were evaluated at 1.5, 2.5, and 4.5 μM for midazolam and 1-hydroxymidazolam, and at 1.5, 3.5, and 7.0 μM for nifedipine and dehydronifedipine. Intrarun precision and accuracy were assessed by analyzing five replicates at each concentration (*n* = 5), and the inter-run assays were performed in three consecutive runs (*n* = 15). Results were expressed as the relative standard deviation (RSD%) for precision and the relative error (RE%) for accuracy.

Selectivity was investigated by injecting samples of extract solutions and liver microsome solutions under the same experimental conditions to assess potential interfering peaks at the same retention times as the analytes. To evaluate the carryover, two samples of methanol/water (1:1) were injected before and after the injection of the analyte solution at the upper limit of quantification and the IS at the working concentration.

### Effect of *M. ilicifolia* Extracts on CYP3A4 Activity

Human liver microsomes are commonly applied to evaluate drug interactions mediated by the cytochrome P450 enzymes. The approach involves experiments to evaluate the potential of a test compound to inhibit CYP450 enzymes. For the initial screening, specific substrate and inhibitor (positive control) of the CYP450 isoform are incubated with the microsomes and a NADPH regeneration solution in the presence or absence of the test compound. Subsequently, the concentrations of the parent markers and their metabolites are determined, and the remaining activity of the enzyme can be calculated by comparison with the control.^19^ Specifically for the CYP3A4, the midazolam hydroxylation and the oxidation of nifedipine to dehydronifedipine are considered reliable markers.^20^

### Screening of Potential CYP3A4 Inhibitors

To evaluate the influence of *M. ilicifolia* extracts on hepatic metabolism mediated by the CYP3A4 isoform, the substrates midazolam (433 μM) and nifedipine (563 μM) were incubated in microsomal medium in the presence of the extracts. The influence of the main secondary metabolites in *M. ilicifolia* (catechin, epicatechin, quercetin, kaempferol, and friedelin) was also evaluated.

The solution containing human liver microsomes was prepared immediately before the experiment. The microsomal pool at 20 mg/mL was diluted in 100 mM phosphate buffer (pH 7.4), resulting in a protein concentration of 0.4 mg/mL for the midazolam study and 0.6 mg/mL for the nifedipine study. The NADPH regeneration system was prepared by mixing 600 μL of glucose-6-phosphate (50 mmol/L), 600 μL of NADP^+^ (2.5 mmol/L), and 300 μL of glucose-6-phosphate dehydrogenase (8 units/mL) solutions, all prepared in Tris–KCl buffer.

The screening study evaluated the potential inhibition of the five extracts (AE, HAE1, HAE2, HEE, and CDE) at 2 mg/mL, as well as the compounds catechin, epicatechin, quercetin, kaempferol, and friedelin, each at 100 μM. Ketoconazole at 16 μM, a known CYP3A4 enzyme inhibitor, was used as a positive control.

The incubation medium consisted of 50 μL of the microsomal solution, 2.5 μL of the substrate solution (midazolam or nifedipine), 95 μL of 100 mM phosphate buffer pH 7.4, and 2.5 μL of the sample solution (*M. ilicifolia* extract, secondary metabolites, or ketoconazole). For the negative control, 2.5 μL of acetonitrile/water 1:1 (v/v) was used. The medium was preincubated at 37 °C for 5 min. The reaction was initiated by adding 50 μL of the NADPH regeneration system. The incubation times for the midazolam and nifedipine assays were 10 and 15 min, respectively. After the incubation, 1000 μL of ethyl acetate was added to stop the reaction. Then, 25 μL of the alprazolam (IS) working solution was added.

The samples were subjected to a liquid–liquid extraction with ethyl acetate. For the midazolam study, 100 μL of a 0.5 mol/L sodium hydroxide solution was added. The samples were shaken at 500 rpm for 30 min and then centrifuged at 1600*g* at 4 °C for 10 min. An aliquot of 850 μL of the organic phase was collected and evaporated by using a sample concentrator. Finally, the samples were reconstituted in 100 μL of mobile phase, and 50 μL were injected for HPLC analysis. The response of the negative control represented 100% activity. Therefore, the percentage of remaining activity (%RA) was calculated by comparing the concentration of metabolites formed in the samples containing the potential inhibitors to that in the control samples. Inhibition was considered significant when the %RA was less than 50%.

### Determination of IC_50_ of *M. ilicifolia* Extract

The HAE1 was selected for the IC_50_ assay and diluted to concentrations of 0.25, 0.5, 1.0, 2.0, and 4.0 mg/mL (*n* = 3). These solutions were added to the microsomal medium and incubated with the probe substrates, midazolam or nifedipine. Negative control samples were also included in the analysis. After the incubation procedure, the samples were subjected to liquid–liquid extraction and analyzed by HPLC to quantify the metabolites 1-hydroxymidazolam, and dehydronifedipine. The %RA was calculated as previously described, and the IC_50_ value for the HAE1 was determined according to the Hill equation using the GraphPad Prism 8 software (GraphPad Software, San Diego, USA):

1 $$Y=\frac{100}{1+10(\log{\mathrm{IC}}_{50}-X)\times \lambda }$$

where *Y* is the normalized percentage of inhibition, *X* is the inhibitor concentration, and λ is the Hill factor.

### Effect of *M. ilicifolia* Extracts on P-gp Activity Using the Caco-2 Cell Model

#### Caco-2 Cell Culture

Caco-2 cells from the Adolfo Lutz Institute (São Paulo, Brazil) were maintained in DMEM supplemented with glucose (4.5 g/L), 10% fetal bovine serum, 1% nonessential amino acid solution, 1% glutamine solution (200 mM), and 2.2 g/L sodium bicarbonate. The cells were kept at 37 °C in an atmosphere of 90% relative humidity and 5% CO_2_. The medium was replaced every 48 h. Once 80–90% confluence was reached, the cells were transferred to new flasks containing DMEM, in order to obtain a transfer ratio of 1:4. To perform the permeability study, cells were seeded at a density of approximately 5 × 10^4^ cells/cm^2^ in Transwell plates (Corning, Lowell, USA) composed of 12 wells with a polycarbonate membrane, porosity of 0.4 μm, and area of 1.12 cm^2^. Cell culture on the plates was performed by adding 0.5 mL of DMEM to the apical portion and 1.5 mL of the same medium to the basolateral portion. The media were replaced every 48 h, until completing 21 days.

### Permeability Study

Before the permeability study, the integrity of the cell monolayer was checked by measuring transepithelial electrical resistance using a Millicell-ERS voltmeter (Millipore Corp, Bedford, MA, USA). Cells were considered confluent when resistance values were higher than 350 mΩ cm^2^.

The experiment was conducted with four plates: two plates (1 and 2) for evaluating transport from the apical to the basolateral side (A–B), and two plates (3 and 4) for evaluating transport from the basolateral to the apical side (B–A). Fexofenadine (50 μM) was used as a substrate marker of the P-gp activity. The potential inhibitors tested were HAE1 solutions (200, 150, 100, 50, and 25 μg/mL), epicatechin (100 μM) and verapamil (100 μM) as a positive control. The cells were washed with HBSS buffer, and the solutions were aspirated. During the preincubation step, 1.5 mL of HBSS pH 7.4 was added to the basolateral side and 0.5 mL of the inhibitor solution to the apical side. In the control plates, 0.5 mL of HBSS at pH 6.0 with 1% dimethyl sulfoxide (DMSO) was added. The plates were preincubated at 37 °C with shaking for 30 min.

Then, the solutions from the apical side of plates 1 and 2 were removed and replaced with solutions containing the inhibitor plus fexofenadine (50 μM) in HBSS pH 6.0. In plates 3 and 4, the solution from the basolateral side was aspirated and replaced with the fexofenadine solution (50 μM) in HBSS pH 7.4. The plates were incubated at 37 °C while being shaken at 50 rpm. After 30, 60, 90, 120, and 180 min, 200 μL of the solutions were collected from the basolateral side of plates 1 and 2, and from the apical side of plates 3 and 4. Each well was replenished with the same solution, maintaining a constant volume.

The collected samples were analyzed for the fexofenadine content. Briefly, the HPLC analyses were carried out on a Waters Alliance system (New Castle, USA), composed of a quaternary pump, autosampler, fluorescence detector, and Empower software. Separation was achieved using a Zorbax Eclipse Plus C18 column (150 mm × 4.6 mm, 5 μm) from Agilent (Santa Clara, USA), at 40 °C. The mobile phase consisted of phosphate buffer (pH 3.2) with 5 mM sodium heptane sulfonate and acetonitrile (60:40), with a flow rate of 1 mL/min. Fluorescence detection was used to monitor the analytes (λ_exc_ = 230 nm; λ_em_ = 297 nm).

The apparent permeability coefficient (*P*_app_) was calculated based on the fexofenadine transport (cm/s), measured by the flow of fexofenadine through the cell membrane, according to the following equation:^21^

2 $$P_{\mathrm{app}}=\frac{\Delta Q}{\Delta T\times A\times C_{o}}$$

where Δ*Q*/Δ*T* is the amount of drug (ng/min) in the receptor compartment as a function of time, obtained from the slope of the linear portion of the transported amount versus time in the graph; *C*_o_ is the initial concentration of fexofenadine in the donor compartment (ng/mL); *A* is the area of the permeable cell culture support.

IC_50_ was calculated using the Hill equation and GraphPad Prism 8 software (GraphPad Software, San Diego, USA). For this, the apparent permeability responses (*P*_app_(B–A)) were transformed into % control, according to the equation:^22^

3 $$\%\;control=\frac{P_{app}\left(B\text{−}A\right)\;with\;inhibitor}{P_{app}\left(B\text{−}A\right)\;with\;out\;inhibitor}$$

## Results and Discussion

### Content of Total Phenolics, Tannins, and Flavonoids

Since the magnitude of the clinical effect depends on the concentration of the phytocompounds, which can vary according to the cultivars, growing conditions, the extraction process, seasonal differences, and harvest, the contents of the total phenolics, tannins, and flavonoids were determined to avoid any bias and to facilitate comparison with previous and future studies. The contents of the secondary metabolites found in the five extract samples are presented in Table 1. The HAE1 exhibited the highest concentration of total phenolics and flavonoids. Regarding tannins, the extract with the highest concentration was achieved in the HAE2, indicating that turboextraction with acetone and water (70:30) allows greater extraction of these secondary metabolites. The CDE showed the lowest concentration of all of the analyzed compounds.

**Table 1:** Content of Total Phenolics, Tannins, and Flavonoids (%; w/w) in *M. ilicifolia* Extracts (*n* = 3)^a^

extract	total phenolics (%)	tannins (%)	flavonoids (%)
AE	6.69 ± 0.10	2.20 ± 0.05	1.15 ± 0.14
HAE1	11.46 ± 0.52	3.36 ± 0.03	2.64 ± 0.17
HAE2	6.36 ± 0.40	3.95 ± 0.07	1.68 ± 0.11
HEE	7.23 ± 0.34	2.17 ± 0.09	2.46 ± 0.12
CDE	1.76 ± 0.46	1.67 ± 0.09	0.21 ± 0.01

a Data are expressed as mean ± standard deviation.

The obtained results are consistent with previous studies investigating the content of phenolic compounds in extracts of *M. ilicifolia*. Martins et al.^23^ found 8.72% (w/w) of total phenolics and 3.11% (w/w) of tannins in extracts obtained by aqueous infusion of the leaves. Mossi et al.^24^ reported total tannin levels ranging from 7.42 to 29.20% (w/w) in samples of *M. ilicifolia* from different locations, with the highest tannin levels correlating with higher average annual temperatures. The relation between high incidence of light and greater production of phenolic compounds in *M. ilicifolia* was also described by Radomski and Bull,^25^ which found total phenolic contents ranging from 8.0 to 19.3% (w/w) and tannin contents from 3.5 to 12.7% (w/w) in AE. Rocha et al.^26^ also analyzed AE of *M. ilicifolia* leaves, finding polyphenolic contents of 7.16% (w/w) for shade-grown samples and 10.29% (w/w) for samples grown in full sun. A previous study demonstrated that plants exposed to full sun produce tannins as a defense mechanism against ultraviolet rays, as these compounds may absorb excess radiation.^27^ Such variability poses a challenge when predicting potential clinical interactions with plant-derived medicines, highlighting the need to quantify the potential modulators for comparing different studies.^28^

### Development and Validation of the HPLC Method

An HPLC method was developed for the identification and quantification of midazolam and nifedipine and their metabolites, 1-hydroxymidazolam and dehydronifedipine, respectively. The concentrations of these two drugs, as well as their metabolites, are used as markers of CYP3A4 enzymatic activity. Thus, a selective and reliable quantitative method for determining these compounds during the experiments to evaluate CYP3A4 inhibition in human liver microsomes is required. In this study, an HPLC analytical method was developed and validated for the simultaneous quantification of midazolam, nifedipine, 1-hydroxymidazolam and dehydronifedipine. The method successfully separated these four analytes and alprazolam (IS) with adequate resolution (Figure 1).

**Figure 1:**
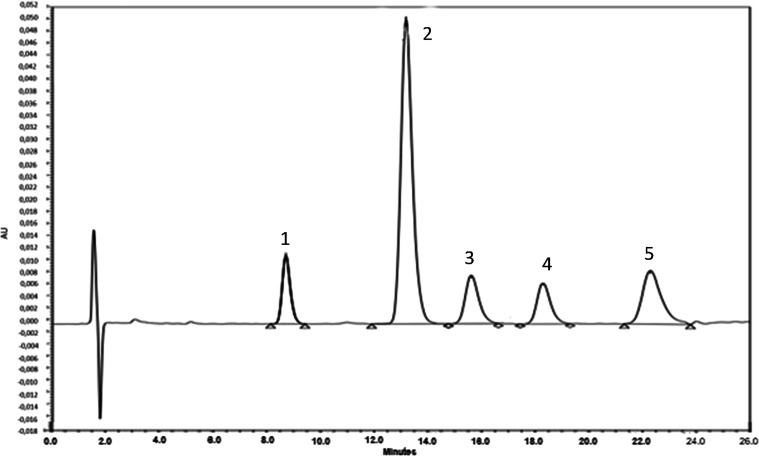
Chromatogram obtained by the developed high-performance liquid chromatography method for (1) dehydronifedipine 0.5 μM (8.7 min), (2) alprazolam (IS) 15 μM (13.2 min), (3) nifedipine 0.5 μM (15.6 min), (4) 1-hydroxymidazolam 0.5 μM (18.4 min), and (5) midazolam 0.5 μM (22.3 min).

All analytical curves showed a linear relationship between the concentration and the analytical signal (analyte peak area/IS peak area). Statistical analysis confirmed that the assumptions of normality, homoscedasticity, and independence of the residuals were met. Additionally, ANOVA (α = 0.05) verified both the significance of the regression and the absence of deviations from linearity for all analytes. The correlation coefficients were 0.9958, 0.9969, 0.9951, and 0.9989 for midazolam, 1-hydroxymidazolam, nifedipine, and dehydronifedipine, respectively, proving the linearity of the method.

RSD and RE values obtained during method validation are presented in Table 2. These values indicate that the developed method demonstrates adequate precision and accuracy for quantifying the analytes within the employed concentration range. Furthermore, the established limit of quantitation was 0.5 μM for all of the analytes.

**Table 2:** Precision (Relative Standard Deviation—RSD%) and Accuracy (Relative Error—RE%) Results Obtained for Midazolam, 1-Hydroxymidazolam, Nifedipine, and Dehydronifedipine by the Developed High-Performance Liquid Chromatographic Method

		precision (RSD%)		accuracy (RE%)	
compound	concentration (μM)	intrarun (*n* = 5)	interrun (*n* = 15)	intrarun (*n* = 5)	interrun (*n* = 15)
midazolam	0.5	3.48	3.00	–5.27	–5.48
	1.5	5.09	6.97	1.76	–4.31
	2.5	3.51	7.24	–5.35	0.48
	4.5	2.34	9.74	–6.56	–1.19
1-hydroxymidazolam	0.5	2.75	2.78	11.92	9.37
	1.5	3.17	6.68	4.98	–2.54
	2.5	5.02	7.52	–6.99	0.26
	4.5	7.21	7.58	–8.44	–6.47
nifedipine	0.5	3.34	2.97	10.28	10.37
	1.5	4.22	7.05	3.29	1.48
	3.5	5.31	8.40	–11.77	–5.58
	7.0	2.68	4.23	–4.27	–4.95
dehydronifedipine	0.5	0.85	1.22	13.38	13.10
	1.5	2.75	7.03	–7.91	0.17
	3.5	5.10	7.56	–7.37	–0.99
	7.0	1.01	2.95	–2.14	–1.84

The method proved to be selective, since no interfering peaks were observed in the chromatograms at the same retention time of the analytes in the samples containing the liver microsomes, *M. ilicifolia* extracts, catechin, epicatechin, quercetin, kaempferol, friedelin, and ketoconazole were injected. In the chromatograms of the blank solution (methanol/water; 1:1 v/v) obtained before and after the injection of the analytes at the upper limit of quantification, no peak corresponding to interferences was observed, indicating no residual effect.

### Effects of *M. ilicifolia* Extracts on CYP3A4 Activity

An inhibition screening study was performed to identify which extracts or secondary metabolites of *M. ilicifolia* might potentially inhibit CYP3A4 enzymes. Remaining activity of less than 50% may indicate significant inhibition potential.^29^ Since CYP3A4 enzymes have multiple substrate binding sites, two different CYP3A4 substrates (midazolam and nifedipine) were used to precisely evaluate the potential for drug interactions with this enzyme.^26^ Figure 2 shows the remaining activity (RA%) of all *M. ilicifolia* metabolites and extracts evaluated.

**Figure 2:**
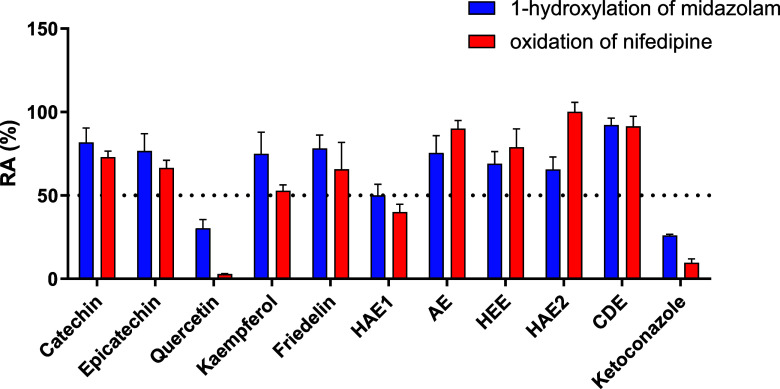
Remaining activity (RA%) of CYP3A, evaluated by the reactions of 1-hydroxylation of midazolam and oxidation of nifedipine, in the presence of extracts (2 mg/mL), the secondary metabolites of *M. ilicifolia* (100 μM), and ketoconazole (16 μM; positive control).

The HAE1 showed a %RA of 50.1% for midazolam 1-hydroxylation and 40.1% for nifedipine oxidation, indicating moderate CYP3A4 inhibition. The other extracts exhibited %RA values higher than 50%. Hence, although these extracts presented some inhibition, it was not considered significant.

Polyphenols have inhibitory effects on CYP3A4 activity by forming covalent bonds that lead to enzyme inactivation. However, in some cases, this inhibition has been shown to be reversible.^13^ Although tannins are an abundant class in *M. ilicifolia*, catechin and epicatechin exhibited %RA values higher than 50% when tested isolated. In previous studies, epicatechin demonstrated 50% inhibitory activity on the CYP3A enzyme assessed by the testosterone 6β-hydroxylation. In the same study, catechin showed an inhibitory effect higher than 50%.^14^

Flavonoids also present inhibitory effects on certain CYP450 forms, including CYP3A4. Quercetin markedly inhibited CYP3A4, both in the 1-hydroxylation of midazolam and in the oxidation of nifedipine, through a reversible and noncompetitive mechanism.^30^ Rastogi and Jana^31^ evaluated the influence of this flavonoid in the 1-hydroxylation of midazolam using human liver microsomes and found an IC_50_ value of 4.30 ± 0.04 μM. In the same study, ketoconazole (positive control) showed an IC_50_ value of 0.06 ± 0.02 μM. Kimura et al.^14^ evaluated the influence of quercetin and kaempferol on the 6β-hydroxylation of testosterone mediated by CYP3A4 and found 100% inhibition for both flavonoids at a concentration of 100 μM. The IC_50_ was 8.8 μM for kaempferol and 22.1 μM for quercetin. The inhibition of the CYP3A4 isoform depends on the chemical structure of the flavonoid, in which the number and position of hydroxyl groups influence the binding mechanism and, therefore, the level and mode of enzymatic inhibition. Flavonoids with many hydroxyl groups are generally considered to be more potent inhibitors of CYP450. However, other factors may also affect the inhibitory potency depending on the substrate and the specific CYP450 isoform.^32^

Subsequently, HAE1 was subjected to a study to determine the IC_50_, which represents the concentration needed to reduce the enzymatic activity by half. The IC_50_ values found for the HAE1 were 0.62 mg/mL (0.52–0.73) for midazolam 1-hydroxylation and 0.43 mg/mL (0.33–0.57) for nifedipine oxidation (Figure 3). The HAE1 presented higher amounts of total phenolics, tannins, and total flavonoids, suggesting that these secondary metabolites are likely associated with the inhibitory activity of *M. ilicifolia*. Our previous study demonstrated that HAE1 provided the highest levels of all the assayed secondary metabolites: catechin (0.49% w/w), epicatechin (0.79% w/w), quercetin (0.01% w/w), kaempferol (0.004% w/w), and friedelin (0.98% w/w).^10^

**Figure 3:**
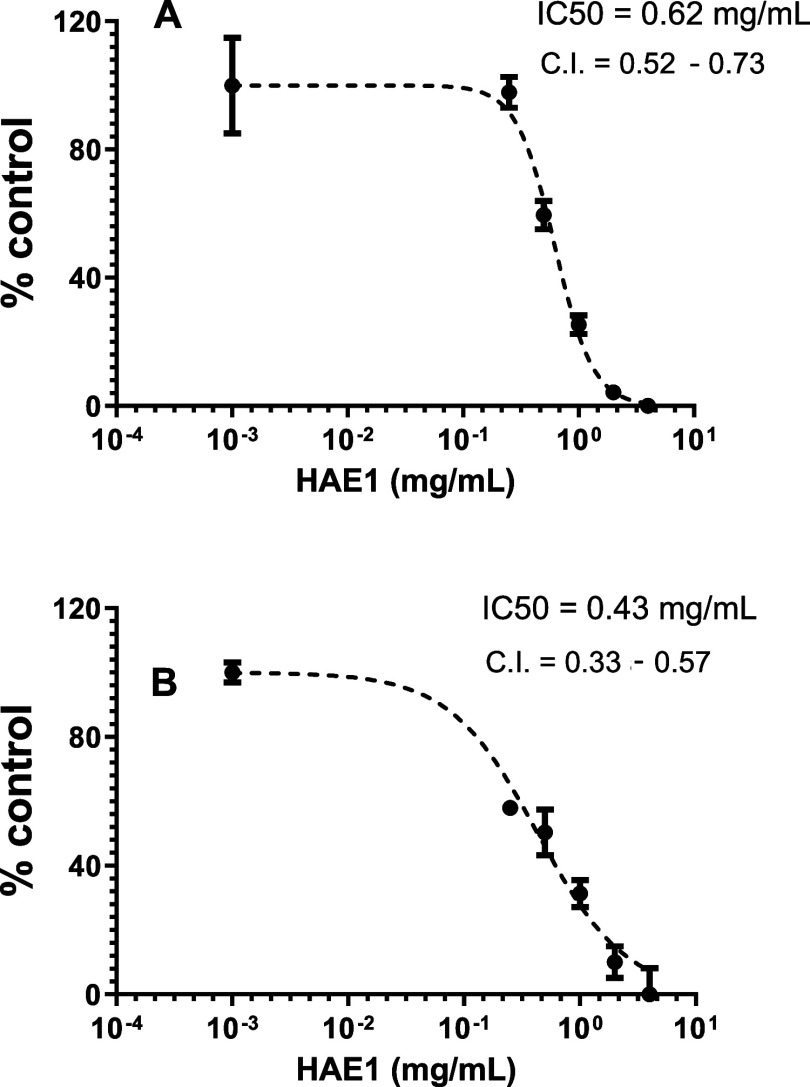
Determination of IC_50_ and confidence interval (CI) of hydroacetonic extract (HAE1) on CYP3A inhibition. (A) 1-Hydroxylation of midazolam and (B) oxidation of nifedipine (*n* = 3).

This study demonstrated that the HAE of *M. ilicifolia* inhibited CYP3A4 in a human *in vitro* model, confirming our previous findings in rats. Since CYP3A4 plays a critical role in the metabolism of up to 50% of drugs that undergo biotransformation, this evidence highlights the potential for herb–drug interactions in humans. Such interactions could increase the risk of toxic or undesirable effects in drugs affected by CYP3A4 inhibition.^33^

### Effect of *M. ilicifolia* Extracts on P-gp Activity

During the permeability study, the flux of fexofenadine through Caco-2 cell monolayers was linear for up to 3 h in both the apical–basolateral (A–B) and basolateral–apical (B–A) directions. The *P*_app_(B–A) of fexofenadine (53.53 × 10^–6^ cm/s) was approximately 8-fold higher than that *P*_app_(A–B) (6.38 × 10^–6^ cm/s). The obtained efflux ratio (8.39) was higher than 2.0, proving that fexofenadine is a substrate for apical efflux transporters, such as P-gp (Table 3).^34^ The use of verapamil in the experiment decreased the fexofenadine efflux ratio (1.64), indicating a reduction in the P-gp-mediated efflux mechanism, since verapamil is a known inhibitor of this transporter.^35^

**Table 3:** Apparent Permeability Coefficients—*P*_app_(A–B) and *P*_app_(B–A)—and Efflux Ratio of Fexofenadine in the Absence (Control) and Presence of Verapamil, Hydroacetonic Extract (HAE1), and Epicatechin (*n* = 3)

sample	*P*_app_(A–B) (cm/s)	*P*_app_(B–A) (cm/s)	efflux ratio
control	6.38 × 10^–6^	53.53 × 10^–6^	8.39
verapamil	18.70 × 10^–6^	30.68 × 10^–6^	1.64
HAE1	19.06 × 10^–6^	32.67 × 10^–6^	1.71
epicatequin	6.16 × 10^–6^	28.33 × 10^–6^	4.60

Epicatechin, a secondary metabolite of *M. ilicifolia*, reduced the *P*_app_(B–A) of fexofenadine and decreased the efflux rate, indicating a slight inhibitory potential. Previous studies have reported controversial results regarding the action of epicatechin on P-gp. Kitagawa et al.^36^ did not observe any effect of epicatechin on P-gp activity in KB-C2 cells. Another study demonstrated that (−)­epicatechin, despite inhibiting the transport of rhodamine, a P-gp substrate, was also able to increase the active transport of LDS, a fluorescent P-gp substrate.^37^ The P-gp inhibitory effect of catechins seems to be influenced by their chemical structure. In particular, the presence of a galloyl group on the C-ring and the trihydric pyrogallol group on the B-ring appear to play important roles in the inhibition.^36^ Furthermore, other secondary metabolites identified in *M. ilicifolia*, such as quercetin, kaempferol, and friedelin, exhibited inhibitory activity on P-gp, mainly by reducing the expression and function of this transporter.^33,38,39^

The HAE1 (200 μg/mL) led to an approximately 3-fold increase in the *P*_app_(A–B) of fexofenadine, and a 1.6-fold reduction in *P*_app_(B–A) (Table 3). Similar to verapamil, HAE1 promoted a reduction in the fexofenadine efflux ratio, indicating an inhibitory effect on the efflux of fexofenadine by P-gp. A previous study demonstrated that the HAE of *M. ilicifolia* promoted a 2.5-fold increase in the intracellular concentration of fexofenadine compared to the control group, supporting the potential inhibition of this extract on the action of P-gp.^16^

The HAE1 was tested at five concentrations to obtain the IC_50_ value (20.42 μg/mL), as presented in Figure 4. This value is relatively low compared to the doses of *M. ilicifolia* extracts used in clinical practices. These findings suggest a potential inhibition of P-gp activity by the HAE1, leading to increased absorption of drugs that are substrates of this protein, when concomitantly administered.^40,41^

**Figure 4:**
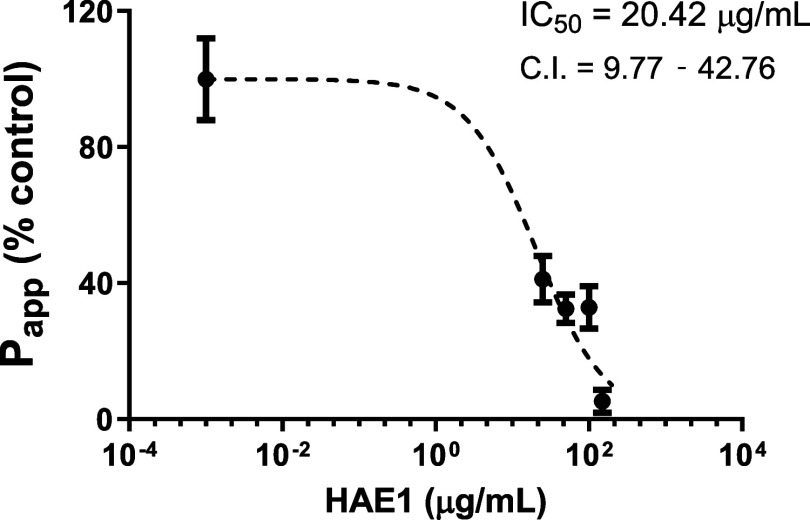
Determination of IC_50_ of hydroacetonic extract (HAE1) on P-glycoprotein inhibition based on the transport of fexofenadine in the secretory direction (B–A) through Caco-2 cells (*n* = 3).

On the other hand, inhibition of P-gp may be useful in cases of tumor cell resistance to chemotherapeutic drugs. This resistance is one of the main difficulties with respect to the success of chemotherapy and may lead to a lack of response to treatment in cases of metastatic cancer. The primary mechanism of resistance is related to the presence of P-gp on the surface of the tumor cells, which actively pumps out the chemotherapeutic drug. Thus, future studies using HAE of *M. ilicifolia* as an adjuvant to overcome the tumor resistance against chemotherapeutic agents would contribute to further explore the benefits and safety of this species.^42,43^

## Conclusions

The present study provided new information about the potential of *M. ilicifolia* to cause pharmacokinetic interactions with substrate drugs of CYP enzymes and the P-gp transporter. The coadministration of *M. ilicifolia* extracts with substrate drugs of CYP3A4 and P-gp may lead to increased plasma concentrations and consequently to toxic effects, especially when the substrate drug has a low therapeutic index. The limitations of the present findings are mainly related to the correlation with the clinical response during the treatment of a patient who is taking *Maytenus* products concomitantly. Therefore, further studies should be conducted to investigate the clinical relevance of these interactions, besides establishing the mechanisms of action and identifying the compounds related to the inhibition.

## Abbreviations

AE: aqueous extract
ANOVA: analysis of variance
AUC: area under the curve
CDE: commercial dry extract
*C*_max_: maximum plasma concentration
DMEM: Dubelco’s modified Eagle’s medium
HAE1: hydroacetonic extract 1
HAE2: hydroacetonic extract 2
HEE: hydroethanolic extract
HDI: herb–drug interactions
HPLC: high-performance liquid chromatography
IS: internal standard
NADP^+^: nicotinamide adenine dinucleotide phosphate sodium salt
*P*_app_: apparent permeability coefficient
P-gp: P-glycoprotein
RA: remaining activity
RE: relative error
RSD: relative standard deviation
TEER: transepithelial electrical resistance
UV–vis: ultraviolet–visible
